# *Toxoplasma gondii* and *Rickettsia* spp. in ticks collected from migratory birds in the Republic of Korea

**DOI:** 10.1038/s41598-022-16785-0

**Published:** 2022-07-25

**Authors:** A.-Tai Truong, Mi-Sun Yoo, Subin Min, Ji-Yeon Lim, Hyun-Ji Seo, Heung-Chul Kim, Sung-Tae Chong, Terry A. Klein, Chang-uk Park, Sook-Young Cho, Chang-Yong Choi, Young-Soo Kwon, Miran Kim, Soon-Seek Yoon, Yun Sang Cho

**Affiliations:** 1 Parasitic and Honeybee Disease Laboratory, Bacterial Disease Division, Department of Animal and Plant Health Research, Animal and Plant Quarantine Agency, Gimcheon-si, Gyeongsangbuk 39660 Republic of Korea; 2 Faculty of Biotechnology, Thai Nguyen University of Sciences, Thai Nguyen, 250000 Vietnam; 3 Force Health Protection and Preventive Medicine, Medical Department Activity-Korea, 65th Medical Brigade, Unit 15281, APO AP, 96271-5281 USA; 4 U Inc. 34-gil, Daesakwan-ro, Yongsan-gu, Seoul, 04409 Republic of Korea; 5 Migratory Bird Research Center, Korea National Park Research Institute, Korea National Park Service, Sinan County, Jeollanam 58863 Republic of Korea; 6 Department of Agriculture, Forestry, and Bioresources, Seoul National University, Gwanak-gu, Seoul, 08826 Republic of Korea

**Keywords:** Microbiology, Diseases

## Abstract

Migratory birds disperse ticks and associated tick-borne pathogens along their migratory routes. Four selected pathogens of medical importance (*Coxiella burnetii, Rickettsia* spp., *Francisella tularensis,* and *Toxoplasma gondii*) were targeted for detection in 804 ticks (365 pools) collected from migratory birds at Hong and Heuksan Islands in the Republic of Korea (ROK) from 2010 to 2011 and 2016. *Toxoplasma gondii* and *Rickettsia* spp., were detected in 1/365 (0.27%) and 34/365 (9.32%) pools of ticks, respectively. *T. gondii* and five rickettsial species were recorded in ticks collected from migratory birds for the first time in ROK. The five rickettsial species (*R. monacensis, Candidatus* Rickettsia longicornii*, R. japonica, R. raoultii*, and *R. tamurae*) were identified using sequence and phylogenetic analysis using *ompA* and *gltA* gene fragments. *Rickettsia* spp. are important pathogens that cause rickettsiosis in humans, with cases recorded in the ROK. These results provide important evidence for the potential role of migratory birds in the introduction and dispersal of *T. gondii* and *Rickettsia* spp. along their migratory routes and raise awareness of potential transmission of zoonotic tick-borne pathogens associated with migratory birds in the ROK.

## Introduction

Migratory birds may play an important role in the potential spread of ticks and associated tick-borne pathogens. Ticks that feed on birds are transported across geographical barriers to new habitats along their migratory routes^1–3^. In addition, ticks harboring tick-borne pathogens are likely dispersed to different regions during the annual migration of birds^2,4,5^. Tick infestation levels are dependent upon ground-feeding behavior and movement characteristics of birds^6^. While resident ground feeding birds may be more heavily infested with ticks, migratory birds may play a more significant role in the long-distance dispersal of tick species and associated pathogens^1^. Environmental and climate changes may provide unexpected opportunities for the potential introduction of ticks and associated pathogens^7–9^. Therefore, information on migration routes of birds is important to understand the potential influence of migratory birds in the future distribution of ticks and tick-borne pathogens along their migration routes, and to raise awareness for the potential transmission of tick-borne pathogens of medical importance in the Republic of Korea (ROK).

Birds are natural reservoirs of selected tick-borne pathogens that are of veterinary and medical importance^10,11^. Evidence of various tick-borne pathogens harbored by ticks infesting migratory and resident birds has been shown worldwide. *Borrelia* and *Rickettsia* spp. were the most prevalent tick-borne microorganisms detected in *Ixodes* spp. collected from migratory birds from European countries^12–14^, USA^15,16^, and Asia^17,18^. *Rickettsia* spp. were also detected from *Hyalomma* spp. that originated from African countries and were transported by migratory birds to Italy^5^. Meanwhile, *Haemaphysalis* spp. that infested birds were identified that harbor *Rickettsia* spp., *Borrelia burgdorferi*, *Anaplasma* spp., and *Ehrlichia* spp.^16,18^, and also possibly contributed to the dispersal of Severe Fever with Thrombocytopenia Syndrome (SFTS) virus present in China, Japan, and Korea^19^. Consequently, identification of tick species and associated tick-borne pathogens and host migratory birds are important to assess the potential risk of tick-borne disease introductions in each region where there are suitable habitats for migratory birds.

In the ROK, Heuksan-do (do = island), Hong-do, and Nan-do are stopover habitats of migratory birds that are located in the Yellow Sea. Ticks collected from migratory birds were identified to species, that included eight species (*Haemaphysalis flava*, *H. formosensis*, *H. longicornis*, *H. concinna*, *H. ornithophila, Ixodes nipponensis*, *I. turdus*, and *Amblyomma testudinarium*) belonging to three genera. Of the eight species, *I. turdus* and *H. flava* were the most prevalent species collected^20–22^. Only three tick-borne microorganisms (*Borrelia* spp., *A. phagocytophilum,* and *Bartonella grahamii*) were detected in these ticks. *Borrelia* spp. were the most prevalent microorganisms detected in *I. turdus* and *H. flava*, while *A. phagocytophilum* and *B. grahamii* were detected only in *I. nipponensis* and *I. turdus*, respectively^21,22^. However, information for other tick-borne microorganisms such as *Coxiella burnetii*, *Rickettsia* spp., *Toxoplasma gondii*, and *Francisella tularensis* in ticks collected from migratory birds remain unknown. Infections of these pathogens in humans in the ROK were recorded and potentially influence public health^23–28^.

Accordingly, this study aimed to extend our previous work^22^ to survey for the presence of four tick-borne pathogens, *C. burnetii*, *T. gondii*, *F. tularensis,* and *Rickettsia* spp., in ticks collected from migratory birds at two islands, Hong-do and Heuksan-do, ROK. Sequencing and phylogenetic analysis were done for species identification of *Rickettsia* spp.

## Results

### Tick-borne microorganisms in bird ticks

A total of 804 ticks belonging to three genera and seven species were placed in 365 pools according to bird host, date and location of collection, and stage of development (Table 1). *I. turdus* was the most commonly collected species and accounted for 72.89% of all collected ticks*,* followed by *H. flava* with 16.17%*, I. nipponensis* 5.85%, *H. longicornis* 4.23%, *H. phasiana* 0.50%, *H. formosensis* 0.25%, and *A. testudinarium* 0.12% (Table 1).

**Table 1:** Detection of tick-borne pathogens from ticks collected from migratory birds in the Republic of Korea.

Tick species	Living stage (Tick No.; pool No.)	Pathogens (positive pool (MIR))			
		*Rickettsia* spp.	*Toxoplasma gondii*	*Francisella tularensis*	*Coxiella burnetii*
*Ixodes turdus*	Larva (409; 109)	0	0	0	0
	Nymph (151; 103)	1 (0.66%)	0	0	0
	Male adult (0; 0)	0	0	0	0
	Female adult (26; 25)	0	1 (3.85%)	0	0
Subtotal	586; 237	1 (0.17%)	1 (0.17%)	0	0
*Haemaphysalis flava*	Larva (52; 19)	2 (3.85%)	0	0	0
	Nymph (78; 50)	3 (3.85%)	0	0	0
	Male adult (0; 0)	0	0	0	0
	Female adult (0; 0)	0	0	0	0
Subtotal	130; 69	5 (3.85%)	0	0	0
*Haemaphysalis longicornis*	Larva (27; 13)	7 (25.93%)	*0*	*0*	*0*
	Nymph (7; 6)	0	0	0	0
	Male adult (0; 0)	0	0	0	0
	Female adult (0; 0)	0	0	0	0
Subtotal	34; 19	7 (20.59%)	0	0	0
*Ixodes nipponensis*	Larva (28; 15)	8 (28.57%)	0	0	0
	Nymph (19; 19)	11 (57.89%)	0	0	0
	Male adult (0; 0)	0	0	0	0
	Female adult (0; 0)	0	0	0	0
Subtotal	47; 34	19 (40.43%)	0	0	0
*Haemaphysalis phasiana*	Larva (2; 1)	0	0	0	0
	Nymph (2; 2)	0	0	0	0
	Male adult (0; 0)	0	0	0	0
	Female adult (0; 0)	0	0	0	0
Subtotal	4; 3	0	0	0	0
*Haemaphysalis formosensis*	Larva (0; 0)	0	0	0	0
	Nymph (2; 2)	1 (50.00%)	0	0	0
	Male adult (0; 0)	0	0	0	0
	Female adult (0; 0)	0	0	0	0
Subtotal	2; 2	1 (50.00%)	0	0	0
*Amblyomma testudinarium*	Larva (0; 0)	0	0	0	0
	Nymph (1; 1)	1 (100.00%)	0	0	0
	Male adult (0; 0)	0	0	0	0
	Female adult (0; 0)	0	0	0	0
Subtotal	1; 1	1 (100.00%)	0	0	0
Total	804; 365	34 (4.23%)	1 (0.12%)	0	0

MIR = [(number of positive pools)/(total number of ticks)] × 100.

Ticks were assayed for selected pathogens, *Rickettsia* spp., *T. gondii, F. tularensis,* and *C. burnetii*. A total of one and 34 pools of ticks were positive for *T. gondii* (Suppl. Fig. S1) and *Rickettsia* spp. (Suppl. Fig. S2), respectively. *T. gondii* was detected only in one adult female *I. turdus* tick collected from the pale thrush, *Turdus pallidus*.

Although *I. nipponensis* was less commonly collected, a higher proportion was positive for *Rickettsia* spp. (55.88%; 19/34 pools), followed by *H. longicornis* (20.59%; 7/34 pools), and *H. flava* (14.71%; 5/34 pools), while one pool each of *I. turdus* (2.94%; 1/34 pools), *H. formosensis* (2.94%; 1/34 pools), and *A. testudinarium* was positive (2.94%; 1/34 pools). *Rickettsia* spp. were not detected in *H. phasiana*. The overall minimum infection rate (MIR) for *Rickettsia* spp. was 4.23%, but was 100%, 50.0%, 40.43%, 20.59%, 3.85%, and 0.17% for *A. testudinarium, H. formosensis, I. nipponensis, H. longicornis, H. flava,* and *I. turdus,* respectively (Table 1).

### Sequencing and phylogenetic analysis

*Toxoplasma gondii* was confirmed by sequence analysis of repetitive DNA fragments from nested conventional PCR (504 bp). Comparison of generated sequences (Suppl. Table S1) with deposited sequences on NCBI databank showed 100% identity with *T. gondii* sequences detected from mice in India (NCBI accession No.: KC607824) and cattle and goats in Iraq (NCBI accession No.: KX963353 and KX963355).

**Figure 1:**
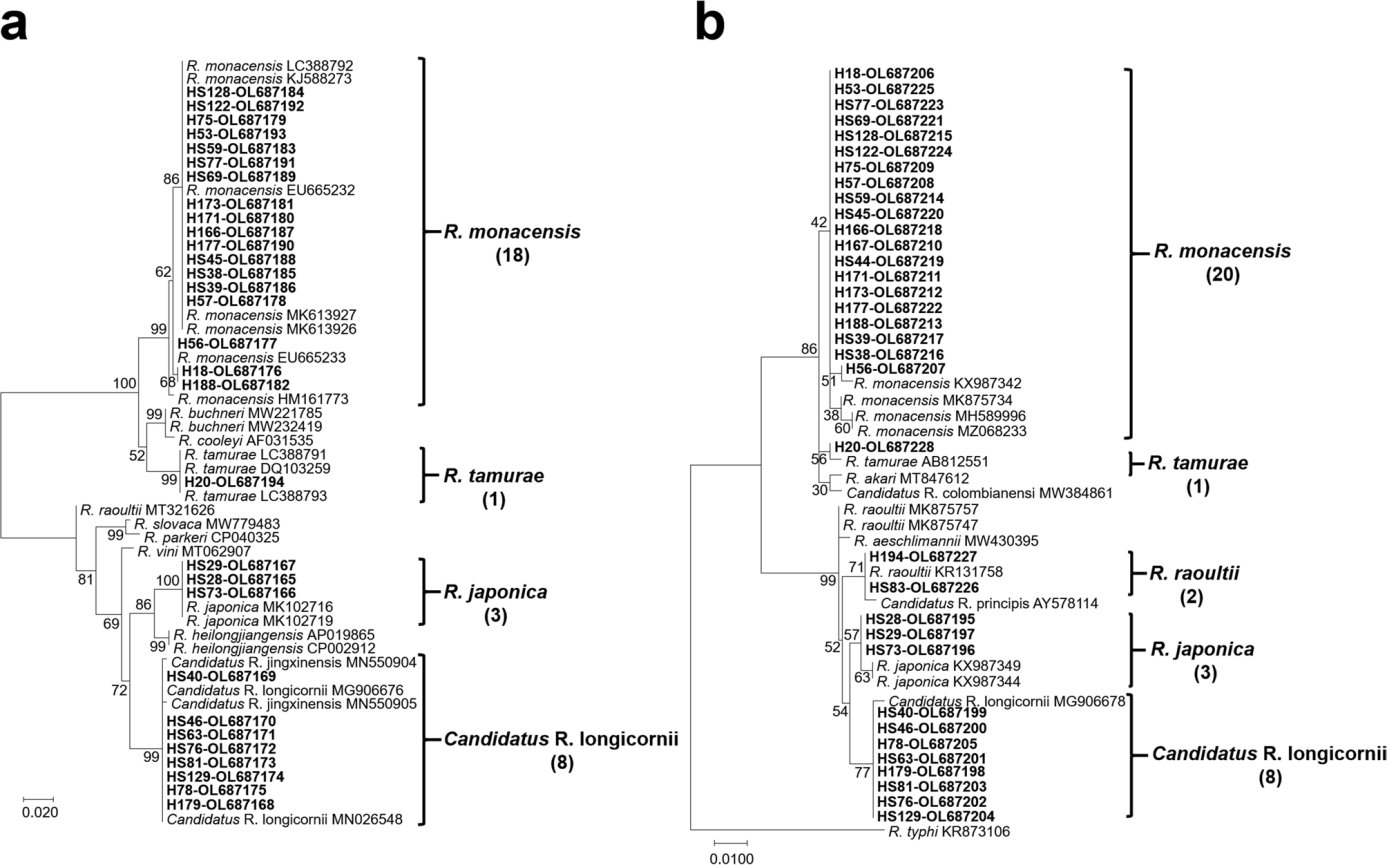
Phylogenetic tree of *Rickettsia* spp. detected from bird ticks. (**a**) Phylogenetic tree based on *ompA* gene fragment sequences (**b**) *gltA* gene fragments showed that *Rickettsia* spp. belonged to five species: *R. monacensis, Candidatus* R. longicornii*, R. japonica, R. raoultii,* and *R. tamurae*. The number of pooled samples for each species is in parentheses. The unique collection/assay number for each tick pool positive for *Rickettsia* spp. and NCBI accession numbers are shown.

Detection of *Rickettsia* spp. targeting *ompA* and *gltA* gene fragments from 34 *Rickettsia* spp. positive tick pools showed that 30 and 34 pools were positive, respectively. The sequences of *ompA* and *gltA* genes were deposited on NCBI with an accession number of each sequence as shown in Table 2 and Suppl. Tables S2 and S3. Variations among the sequences of *ompA* and *gltA* gene fragments were observed. The percent sequence identity among sequences of *ompA* and *gltA* was 78.7% and 93.7%, respectively. Sequences of the *gltA* gene were divided into five *Rickettsia* spp. groups, while the *ompA* gene was separated into four *Rickettsia* spp. The percent identity among the generated sequences for each group ranged from 97.2 to 100.0%. Comparison of generated sequences of the *ompA* and *gltA* gene fragments to the deposited sequences of *Rickettsia* species on NCBI and phylogenetic analysis showed that the detected *Rickettsia* spp. belong to five species (*R. monacensis, Candidatus* Rickettsia longicornii*, R. japonica, R. raoultii,* and *R. tamurae*) with the sequence similarity ranging from 98.80 to 100.00%, while 3 specimens could not be identified to species (Fig. 1; Table 2). Although phylogenetic analysis of *ompA* gene showed that the detected strains (HS40, HS46, HS63, HS76, HS81, HS129, H78, and H179) were in the same clade with *Ca.* R. longicornii and *Ca.* R. jingxinensis (Fig. 1), the sequence analysis showed a higher similarity (100%) of detected strains to *Ca.* R. longicornii than *Ca.* R. jingxinensis (99.2%) (Suppl. Table S2). Therefore, the detected strains were identified as *Ca.* R. longicornii.

**Table 2:** The identified species of *Rickettsia* in bird ticks collected from 2010 to 2011 and in 2016.

No	Group	*Rickettsia* spp.	Tick species	Migratory bird species	Tick pool (GenBank Accession No., *ompA*; *gltA*)
1	I	*R. monacensis*	*Ixodes nipponensis*	Turdus pallidus**	H18 (OL687176; OL687206)
2					H56 (OL687177; OL687207)
3					H57 (OL687178; OL687208)
4				*Emberiza aureola*	H75 (OL687179; OL687209)
5				*Acrocephalus orientalis*	H167 (OL687210*)
6				*E. chrysophrys*	H171 (OL687180; OL687211)
7				*E. spodocephala*	H173 (OL687181; OL687212)
8					H188 (OL687182; OL687213)
9					HS59 (OL687183; OL687214)
10					HS128 (OL687184; OL687215)
11				*Locustella pleskei*	HS38 (OL687185; OL687216)
12					HS39 (OL687186; OL687217)
13					H166 (OL687187; OL687218)
14				*Bradypterus davidi*	HS44 (OL687219*)
15				*L. ochotensis*	HS45 (OL687188; OL687220)
16				*E. rutila*	HS69 (OL687189; OL687221)
17					H177 (OL687190; OL687222)
18				E. elegans**	HS77 (OL687191; OL687223)
19					HS122 (OL687192; OL687224)
20			*I. turdus*		H53 (OL687193; OL687225)
21	II	*Candidatus* Rickettsia longicornii	*Haemaphysalis longicornis*	*E. pallasi*	H179 (OL687168; OL687198)
22				*A. orientalis*	HS40 (OL687169; OL687199)
23					HS46 (OL687170; OL687200)
24				*E. rutila*	HS63 (OL687171; OL687201)
25				*E. chrysophrys*	HS76 (OL687172; OL687202)
26				*E. tristrami*	HS81 (OL687173; OL687203)
27				*E. spodocephala*	HS129 (OL687174; OL687204)
28			*H. flava*	*Phylloscopus inornatus*	H78 (OL687175; OL687205)
29	III	*R. japonica*	*H. flava*	*Tarsiger cyanurus*	HS28 (OL687165; OL687195)
30				*E. chrysophrys*	HS73 (OL687166; OL687196)
31			*H. formosensis*	*Tarsiger cyanurus*	HS29 (OL687167; OL687197)
32	IV	*R. raoultii*	*H. flava*	*E. tristrami*	HS83 (OL687226*)
33				E. elegans**	H194 (OL687227*)
34	V	*R. tamurae*	*Amblyomma testudinarium*	Zoothera aurea**	H20 (OL687194; OL687228)

*Only *gltA* gene was amplified. **These species may be also regarded as residents or partial migrants in Korea, but the birds in this study were all true migrants that were crossing the national borders and ecological barriers like the Yellow Sea.

### Rickettsial infected ticks and migratory bird species

Based on the analysis of the *ompA* and *gltA* gene fragment sequences, *R. monacensis* (58.82%; 20/34 pools) was the most prevalent rickettsial species, followed by *Ca.* R. longicornii (23.53%; 8/34 pools), *R. japonica* (8.82%; 3/34 pools), *R. raoultii* (5.88%; 2/34 pools), and *R. tamurae* (2.94%; 1/34 pool) (Table 2).

*R. monacensis* was detected primarily in *I. nipponensis* (19/20 pools), and one pool of *I. turdus* (1/20). *Ca.* R. longicornii was detected in *H. longicornis*. *R. raoultii* and *R. japonica* were detected in pools of *H. flava. R. tamurae* was detected in *A. testudinarium*. Wild bird species that were hosts of *Rickettsia* infected ticks are shown in Table 2.

## Discussion

*Rickettsia* spp. and *T. gondii* were detected in pools of ticks collected from migratory birds. The results provide additional information about microorganisms harbored by ticks infesting migratory birds in the ROK. A total of five tick-borne microorganisms, including *Borrelia* spp., *A. phagocytophilum, B. grahamii, T. gondii,* and *Rickettsia* spp., have been recorded in ticks collected from migratory birds in the ROK^21,22^*. Rickettsia* spp. and *Borrelia* spp. were the most prevalent tick-borne microorganisms detected from *Ixodes* spp., and the results are consistent with previous reports from other countries^12–16,18^. However, there was a high infection rate and greater species diversity of *Rickettsia* species observed among ticks collected from migratory birds in the ROK compared to reports from other countries. Human infections of *R. monacensis*, *R. japonica,* and *R. raoultii* have been documented in the ROK^27–29^, and *R. tamurae* in Japan^30^. Therefore, ticks from migratory birds likely play a certain role in the transportation of ticks and associated rickettsial pathogens to these islands and the Korean mainland.

*Toxoplasma gondii* has been detected in birds in other areas of the world^31,32^, and the potential role of ticks and migratory birds in dispersing *T. gondii* was suggested^33,34^. However, no evidence of *T. gondii* carried by bird ticks had been previously provided. In this study, one female *I. turdus* tick collected from a pale thrush was positive for *T. gondii*, this is the first report of *T. gondii* detected in *I. turdus* in the ROK. Therefore, the *T. pallidus* and associated ticks may have contributed to the spread of *T. gondii* along its migratory routes*.* Further studies on the presence of *T. gondii* in *T. pallidus* bird and direct transmission of *T. gondii* by *I. turdus* need to be conducted.

Various tick-borne microorganisms (*Rickettsia* spp., *Borrelia* spp., *Anaplasma* spp., *B. grahamii*, and *T. gondii*) were detected in ticks collected from migratory birds in the ROK, of which *Rickettsia* spp. were the most abundant group^20–22^. However, infections of these pathogens in related bird species has not been characterized in the ROK. There are stopover habitats in the ROK for migratory birds on their migration routes between the northeastern Palearctic region, including Russia and eastern China, and southeast Asia^21^. The presence of tick-borne pathogens (*B. garinii*, *A. phagocytophilum*, and *E. chaffeensis*) in migratory birds was confirmed in China^35^. These results suggest that the migratory birds collected in the ROK may be infected and become important natural reservoirs of these tick-borne pathogens. Therefore, it is necessary to conduct further studies on the surveillance of tick-borne pathogens in migratory and resident birds and local tick and animal/bird reservoirs to better understand the role of migratory birds in the potential introduction and spread of tick-borne pathogens.

Wild birds are known to be reservoir hosts of *C. burnetii* and *F. tularensis* and associated ticks might transmit the pathogens to human^36,37^. *Ixodes ricinus* infesting birds were suggested to be the vectors of *C. burnetii*^37,38^. In the ROK, *C. burnetii* and *F. tularensis* were detected more frequently in *H. longicornis* and *H. flava* ticks collected from the environmental habitats and domestic or wild animals^39–41^. However, the two pathogens were not detected in ticks infesting wild birds in this study, and the presence of these two pathogens in ticks feeding on birds in China and other southeast Asian countries located along their migration routes^21^ has not been recorded.

Surveillance of *C. burnetii*, *F. tularensis, Rickettsia* spp., and *T. gondii* in this study demonstrated the presence of *T. gondii* in ticks collected from migratory birds. *Rickettsia* spp., including *R. monacensis, Ca.* R. longicornii*, R. japonica, R. raoultii*, and *R. tamurae*, were the most commonly detected microorganisms in ticks collected from migratory birds. The results provide important information for further studies on the role of migratory birds in dispersion of *T. gondii* and *Rickettsia* spp*.* and raise awareness of tick-borne disease transmission related to migratory birds and associated ticks in the ROK.

## Materials and methods

### Tick collection

Bird and tick surveys were conducted as part of the constant-effort bird banding program of the Migratory Birds Research Center under the National Park Research Institute, Korea National Park Service on islands with access only by government and wildlife capture permits. Ticks were collected from migratory birds at two islands, Hong-do (34° 41′ N, 125° 11′ E) and Heuksan-do (34° 41′ N, 125° 25′ E), Jeollanam Province, ROK, during 2010–2011 and in 2016. These two islands are located in the southwestern tip of the Korean Peninsula, most birds captured in this study were true migrants that were crossing a national border and an ecological barrier, the Yellow Sea.

Tick collected from 2010 to 2011, pooled by species and stage of development, were designated as H1-H195 and in 2016 they were designated as HS1-HS184. Samples in this study were shared with those in the analysis of *Anaplasma* and *Borrelia* species in a previous study^22^, while a few ticks were supplemented to replace destroyed samples for the analysis. Detailed information on collection sites, bird collections, and tick collections were reported in Seo et al.^22^. Ticks were identified using standard morphological keys^43–45^, and then placed in pools according to collection location and date, stage of development, sex, and, host species. Nymphs and larvae were placed in pools of 1–6 and 1–9 ticks, by species and stage of development, respectively, while adult ticks were assayed individually^22^. The pooled samples were placed in 1.5 ml cryovials containing 70% ethanol and stored at – 80 °C until analysis.

### DNA extraction

After washing three times using UltraPure™ DNase/RNase-Free distilled water (Thermo Fisher Scientific, USA), the tick samples were placed in a tissue grinding tube (SNC, Hanam, Korea) containing 0.6 mL phosphate-buffered saline and 2.3 mm stainless-steel beads and then homogenized using Precellys 24 Tissue Homogeniser (Bertin Instruments, Montigny-le-Bretonneux, France). The homogenate was centrifuged at 300×*g* for 1 min and the supernatant was collected for total nucleic acid extraction using Maxwell^®^ RSC Viral Total Nucleic Acid Purification Kits (Promega, USA) and an automated Maxwell RSC Instrument (Promega). The procedure of isolation was done according to the manufacturer’s instructions. Extracted nucleic acids were stored at − 80 °C until further used.

### PCR analysis

Primers and PCR conditions for detection of the selected four targets are shown in Table 3. DNA used for positive control in PCR detection of *C. burnetii* from Nine Mile strain, and of *T. gondii* was from the strain G-P-14-7 that was isolated and stored in Animal and Plant Quarantine Agency, South Korea. Positive control DNA of *F. tularensis* and *Rickettsia* spp. was chemically synthesized according to the sequence information on NCBI with accession No. was CP073128 (*F. tularensis*) and CP047359 (*R. japonica*). Recombinant DNA carrying standard fragments were constructed using the pGEM^®^-T vector system (Promega, Madison, WI, USA) and PCR products amplified by each detection primer pair. Detection of *C. burnetii* was done by two successive PCRs, conventional PCR was performed using primer pair Trans1/2 (Table 3), followed by nested real-time PCR (qPCR) using primer pair Cox111-F/R (Table 3). AccuPower ProFi Taq PCR PreMix (Bioneer, Daejeon, Korea) was used for conventional PCR, each 20 µL reaction mix included: 3 µL DNA template, 1 µL (10 pmol) of each primer, and 15 µL of double-distilled water (ddH_2_O). PCR products obtained by conventional PCR was 250 × diluted and used for nested qPCR. Each 20 µL reaction mixture was composed of 1 µL (10 pmol) of each primer, 1 µL (5 pmol) of probe, 10 µL of PCR premix (IQ supermix, Bio-Rad Laboratories), 2 µL of diluted DNA template, and 5 µL of ddH_2_O. Nested qPCR was performed using the CFX96 Touch Real-time PCR Detection System (Bio-Rad Laboratories, USA). Assays for *F. tularensis* and *T. gondii* were conducted using qPCR. Each 20 µL reaction mix consisted of 3 µL DNA template, 1 µL (10 pmol) of each primer, 1 µL (5 pmol) of probe, 4 µL of ddH_2_O, and 10 µL of PCR premix (IQ supermix, Bio-Rad Laboratories). The sample positive for *T. gondii* using qPCR was used for nested PCR to amplify the repetitive DNA gene fragments (Table 3). After confirming the expected band in electrophoresis agarose gel (1.5%), the PCR product was purified for sequence analysis.

**Table 3:** Primers and PCR conditions for the detection of tick-borne pathogens.

No	Target	Primer name	Sequence (5’-3’)	Target gene	PCR condition	References
1	*Coxiella burnetii*	Cox1111-F	GTC TTA AGG TGG GCT GCG TG	*IS1111*, 295 bp	50 °C (2 min), 95 °C (5 min), 45 cycles of 95 °C (15 s)—60 °C (30 s)	^52^
		Cox1111-R	CCC CGA ATC TCA TTG ATC AGC			
		Probe	FAM-AGC GAA CCA TTG GTA TCG GAC GTT-TAMRA			
		Trans 1	TATGTATCCACCGTAGCCAGTC	*IS1111*, 687 bp	95 °C (5 min), 40 cycles 95 °C (30 s), 57 °C (30 s), 72 °C (1 min)	^53^
		Trans 2	CCCAACAACACCTCCTTATTC			
2	*Francisella tularensis*	Tula4-F	TTACAATGGCAGGCTCCAGA	*Tul4*, 138 bp	95 °C (3 min), 45 cycles 95 °C (15 s)- 60 °C (30 s)	This study
		Tula4-R	TGTCCACTTACCGCTACAGA			
		Tula4-Probe	FAM-TTCTAAGTGCCATGATACAAGCTTCCCA-BHQ-1			
3	*Rickettsia* spp.	ITS-F	GATAGGTCGGGTGTGGAAG	*ITS*, 388 bp	95 °C (3 min), 45 cycles of 95 °C (15 s)–64 °C (15 s)–72 °C (15 s)	^54^
		ITS-R	TCGGGATGGGATCGTGTG			
		RpCS.877p	GGGGGCCTGCTCACGGCGG	*gltA*, 382 bp	95 °C (5 min), 40 cycles 95 °C (30 s), 55 °C (30 s), 72 °C (30 s)	^55^
		RpCS.1258n	ATTGCAAAAAGTACAGTGAACA			
		RpCS.896p	GGCTAATGAAGCAGTGATAA	*gltA*, 338 bp	95 °C (5 min), 40 cycles 95 °C (30 s), 53 °C (30 s), 72 °C (30 s)	
		RpCS.1233n	GCGACGGTATACCCATAGC			
		Rr190k. 71p	TGGCGAATATTTCTCCAAAA	*OmpA*, 650 bp	95 °C (5 min), 40 cycles 95 °C (30 s), 49 °C (30 s), 72 °C (1 min)	^56^
		Rr190k. 720n	TGCATTTGTATTACCTATTGT			
		Rr190k. 71p	TGGCGAATATTTCTCCAAAA	*OmpA*, 532 bp	95 °C (5 min), 40 cycles 95 °C (30 s), 52 °C (30 s), 72 °C (1 min)	
		Rr190k. 602n	AGTGCAGCATTCGCTCCCCCT			^57^
4	*Toxoplasma gondii*	TOXO-F	TCCCCTCTGCTGGCGAAAAGT	*B1*, 98 bp	95 °C (3 min), 40 cycles 95 °C (15 s), 60 °C (30 s)	^58^
		TOXO-R	AGCGTTCGTGGTCAACTATC GATTG			
		Probe	FAM-TCTGTGCAACTTTGGTGTATTCGCAG-TAMRA			
		TOXO4	CGCTGCAGGGAGGAAGACGAAAGTTG	Repeated DNA, 529 bp	95 °C (5 min), 40 cycles 95 °C (30 s), 60 °C (30 s), 72 °C (1 min)	^59^
		TOXO5	CGCTGCAGACACAGTGCATCTGGATT			
		TOXO4	CGCTGCAGGGAGGAAGACGAAAGTTG	Repeated DNA, 504 bp		
		TOXO5-R1	TCTCCTACGCCTCCTCCTCCCTT			This study

For *Rickettsia* spp. detection, qPCR was performed using 2 × Rapi: Detect™ Master mix with dye (SYBR green, Cat. No.: 9799100100; Genesystem, Korea). Each 20 µL reaction mix consisted of 3 µL of DNA template, 1 µL (10 pmol) of each primer, 5 µL of ddH_2_O, and 10 µL of Detect™ Master mix. The positive samples were used for conventional nested PCR targeting two gene fragments (*gltA, ompA*) (Table 3). After confirming the expected band in 1.5% agarose gel by electrophoresis, the nested PCR products were purified and sequenced by Macrogen Inc. (Seoul, Korea).

The Minimum infection rate (MIR) was calculated for each species: MIR = [(number of positive pools)/(total number of tested ticks)] × 100. Each positive pool was estimated to contain only one infected tick^46,47^.

### Sequence and phylogenetic analysis

The generated sequences were compared to the NCBI database using nucleotide Basis Local Alignment Search Tool (BLAST)^48^ for species identification. For analysis of *Rickettsia* spp. the generated sequences of each gene were grouped after alignment using AlignX, a component of Vector NTI Advance v. 10.3 (Invitrogen Co.). Representative sequences of each positive pooled sample were compared to the NCBI database using nucleotide Basis Local Alignment Search Tool (BLAST)^48^. Identical sequences of *Rickettsia* acquired from the NCBI were used for alignment together with generated sequences using Clustal X version 2.0^49^, and Maximum likelihood phylogenetic trees were created using the Kimura 2-parameter model that estimate evolutionary distance based on the nucleotide substitutions^50^, gamma distribution, and bootstrapping 1000 times with MEGA7 software^51^.

### Ethics approval

All field procedures including bird capture, handling, and sampling were under the bird banding station licenses (#501000085200500002, 2011-8, 2016-1, and 2016-2) issued by the local government (Shinan Country), the Korean Ministry of Environment (Yeongsan River Environmental Office), and the Cultural Heritage Administration. This study was approved by The Korea National Park Service (KNPS). Captured birds were safely and ethically examined, sampled, and released safely following the institutional guideline (National Park Research Institute, KNPS) for constant-effort bird banding surveys in Korean National Parks^42^.

## Supplementary Information

Supplementary Information.

## Data Availability

All data generated or analysed during this study are included in this published article (and its Supplementary file). Generated sequences were deposited on NCBI with accession number OL687165–OL687228.
