# Supplementary material for: Toxoplasma gondii and Rickettsia spp. in ticks collected from migratory birds in the Republic of Korea
Source: Sci Rep. 2022 Jul 25;12:12672. doi: 10.1038/s41598-022-16785-0 (PMC9314388; doi:10.1038/s41598-022-16785-0)
Supplement: Supplementary file 1 — Supplementary Information. [file 41598_2022_16785_MOESM1_ESM.docx]

**Supplementary Table S1. DNA sequence of detected *Toxoplasma gondii***

| **Tick pool** | **Sequence (5ꞌ-3ꞌ)** | **Length (bp)** |
| --- | --- | --- |
| **H58** | GCAGGGAGGAAGACGAAAGTTGTTTTTTTAATTTTTTTTCTTTTTGTTTTTCTGATTTTTGTTTTTTTTGACTCCGGCCCAGCTGCCTCTGTCGGGATGAGACCGCGGAGCCGAAGTGCGTTTTCTTTTTTTGATTTTTTTTTGTTTTTTCACAGGCGAGCTCGCCTGTGCTTGGAGCCACAGAAGGGACAGAAGTCGAAGGGGACTACAGACGCGATGCCGCTCCTCCAGCCGTCTTGGAGGAGAGATATCAGGACTGTAGATGAAGGCGAGGGTGAGGATGAGGGGGTGGCGTGGTTGGGAAGCGACGAGAGTCGGAGAGGGAGAAGATGTTTCCGGCTTGGCTGCTTTTCCTGGAGGGTGGAAAAGAGACACCGGAATGCGATCTA | 389 |

**Supplementary Table S2. DNA sequences of *ompA* gene of detected *Rickettsia* spp.**

| **No.** | **Tick pool** | **Sequence (5ꞌ-3ꞌ)** | **Length; NCBI No.** |
| --- | --- | --- | --- |
| **1** | HS28 | AAAATTATTTCAAAAAGCAATACAACAAGGTCTTAAAGCTGCTTTATTCACCACCTCAACCGCAGCGATAATGCTGAGTAGTAGTGGGGCACTCGGTGTTGCTGCAGGTGTTATTGCTACTAATAATGATGCAGCATTTAGTGATGATGCTGATGTCAATAATTGGGATGAGATAACGGCTATAGGGGTAGCTAATGGTCATCCTGCTGGCGGTCCTCAAAACAATTGGGCATTTACTTACGGTGGTGATTATACTATCACTGCAGATGTAGTCGATTGTATTATTAAGGCTATAAATGTTGCGGGTACTAATCCCGTAGGTCTAAATATTGCTCAAAATACCGTCGTTGGTTCGATTATAACGAGAGGTAACTTGTTGCCTGTTACTATTACTGCCGGCAAAAGCTTAACTTTAAATGGTAATAATGCTGTTGCTGCAAATCATGGTTTTGATGCTCCTGCCGATAATTATACAGGTTTAGGAAATATAACTTTAGGGGGA | 502 bp; OL687165 |
| **2** | HS29 | AAAATTATTTCAAAAAGCAATACAACAAGGTCTTAAAGCTGCTTTATTCACCACCTCAACCGCAGCGATAATGCTGAGTAGTAGTGGGGCACTCGGTGTTGCTGCAGGTGTTATTGCTACTAATAATGATGCAGCATTTAGTGATGATGCTGATGTCAATAATTGGGATGAGATAACGGCTATAGGGGTAGCTAATGGTCATCCTGCTGGCGGTCCTCAAAACAATTGGGCATTTACTTACGGTGGTGATTATACTATCACTGCAGATGTAGTCGATTGTATTATTAAGGCTATAAATGTTGCGGGTACTAATCCCGTAGGTCTAAATATTGCTCAAAATACCGTCGTTGGTTCGATTATAACGAGAGGTAACTTGTTGCCTGTTACTATTACTGCCGGCAAAAGCTTAACTTTAAATGGTAATAATGCTGTTGCTGCAAATCATGGTTTTGATGCTCCTGCCGATAATTATACAGGTTTAGGAAATATAACTTTAGGGGGA | 502 bp; OL687167 |
| **3** | HS38 | AAAATTATTTCAAAAGGCAATTCAAAAAGGTCTTAAAACCGCTTTATTCACCACCTCAACCGCAGCATTAATGCTGAGTAGTAGCGGGGCGTTGGGTGTTGCTGCGGGTGTTATTTCTATTAATAGTGCAGCATTTAGTGATCTTGCTGCTGCCGGTAATTGGAATAAGATAACGGCTGGAGGAGTAGCTAATGGTACTTCTGTTGACGGTCCTCAAGACAATAAGGCATTTACTTACGGTGGTCGTCATATTATCACTGCAGATAAAGTCGGTCGTATTATTACGGCTATAAATGTTGCGGCTACTAATCCTATAGGCCTAAATATTGCTGAAAATACCAGCGTCGGTTCTATTGTTACAGATCGTCACTTGTTGCCTGTTAATATTACTGCCGGCAAAAGTTTAACTTTAACCGGTACTGCTGCGTTTGTTCCACGTCATGGTGTTGGTGCTTTTGCTGATACTTATACGGGTTTAGGAAATATAACTTTAGGGGGA | 499 bp; OL687185 |
| **4** | HS39 | AAAATTATTTCAAAAGGCAATTCAAAAAGGTCTTAAAACCGCTTTATTCACCACCTCAACCGCAGCATTAATGCTGAGTAGTAGCGGGGCGTTGGGTGTTGCTGCGGGTGTTATTTCTATTAATAGTGCAGCATTTAGTGATCTTGCTGCTGCCGGTAATTGGAATAAGATAACGGCTGGAGGAGTAGCTAATGGTACTTCTGTTGACGGTCCTCAAGACAATAAGGCATTTACTTACGGTGGTCGTCATATTATCACTGCAGATAAAGTCGGTCGTATTATTACGGCTATAAATGTTGCGGCTACTAATCCTATAGGCCTAAATATTGCTGAAAATACCAGCGTCGGTTCTATTGTTACAGATCGTCACTTGTTGCCTGTTAATATTACTGCCGGCAAAAGTTTAACTTTAACCGGTACTGCTGCGTTTGTTCCACGTCATGGTGTTGGTGCTTTTGCTGATACTTATACGGGTTTAGGAAATATAACTTTAGGGGGA | 499 bp; OL687186 |
| **5** | HS40 | AAAATTATTTCAAAAAGCAATACAACAAGGTCTTAAAGCCGCTTTATTCACCACCTCAACCGCAGCGATAATGCTGAGTAGTAGTGGGGTACTCGGTGTTGCTGCAGGTGTTATTGCTACTAATGATGATGCAGCATTTAGTAATGATGCTGAGGCCAATAATTGGGATGAGATAACGGCTGAAGGGGTAGCTAATGGTATTCCTGCTGGCGGTCCTCAAAACAATTGGGCATTTACTTACAGTGCTGATTATACTATCACTGCAGATGTAGTCGATCGTATTATTACGGCTATAAATGTTGCGGGTACTACTCCCGTAGGTCTAAATATTGCTCAAAATACCGTCGTTGGTTCGATTATAACTAGAGGTAACTTGTTGCCTGTTACTATTGCTGGCAAAAGCTTAACTTTAAACGGTACTAATGCTGTTGCTGCAAATCATGGTTTTGATGCTCCGGCCGATAATTATACAGGTTTAGGAAATATAACTTTAGGGGGA | 499 bp; OL687169 |
| **6** | HS45 | AAAATTATTTCAAAAGGCAATTCAAAAAGGTCTTAAAACCGCTTTATTCACCACCTCAACCGCAGCATTAATGCTGAGTAGTAGCGGGGCGTTGGGTGTTGCTGCGGGTGTTATTTCTATTAATAGTGCAGCATTTAGTGATCTTGCTGCTGCCGGTAATTGGAATAAGATAACGGCTGGAGGAGTAGCTAATGGTACTTCTGTTGACGGTCCTCAAGACAATAAGGCATTTACTTACGGTGGTCGTCATATTATCACTGCAGATAAAGTCGGTCGTATTATTACGGCTATAAATGTTGCGGCTACTAATCCTATAGGCCTAAATATTGCTGAAAATACCAGCGTCGGTTCTATTGTTACAGATCGTCACTTGTTGCCTGTTAATATTACTGCCGGCAAAAGTTTAACTTTAACCGGTACTGCTGCGTTTGTTCCACGTCATGGTGTTGGTGCTTTTGCTGATACTTATACGGGTTTAGGAAATATAACTTTAGGGGGA | 499 bp; OL687188 |
| **7** | HS46 | AAAATTATTTCAAAAAGCAATACAACAAGGTCTTAAAGCCGCTTTATTCACCACCTCAACCGCAGCGATAATGCTGAGTAGTAGTGGGGTACTCGGTGTTGCTGCAGGTGTTATTGCTACTAATGATGATGCAGCATTTAGTAATGATGCTGAGGCCAATAATTGGGATGAGATAACGGCTGAAGGGGTAGCTAATGGTATTCCTGCTGGCGGTCCTCAAAACAATTGGGCATTTACTTACAGTGCTGATTATACTATCACTGCAGATGTAGTCGATCGTATTATTACGGCTATAAATGTTGCGGGTACTACTCCCGTAGGTCTAAATATTGCTCAAAATACCGTCGTTGGTTCGATTATAACTAGAGGTAACTTGTTGCCTGTTACTATTGCTGGCAAAAGCTTAACTTTAAACGGTACTAATGCTGTTGCTGCAAATCATGGTTTTGATGCTCCGGCCGATAATTATACAGGTTTAGGAAATATAACTTTAGGGGGA | 499 bp; OL687170 |
| **8** | HS59 | AAAATTATTTCAAAAGGCAATTCAAAAAGGTCTTAAAACCGCTTTATTCACCACCTCAACCGCAGCATTAATGCTGAGTAGTAGCGGGGCGTTGGGTGTTGCTGCGGGTGTTATTTCTATTAATAGTGCAGCATTTAGTGATCTTGCTGCTGCCGGTAATTGGAATAAGATAACGGCTGGAGGAGTAGCTAATGGTACTTCTGTTGACGGTCCTCAAGACAATAAGGCATTTACTTACGGTGGTCGTCATATTATCACTGCAGATAAAGTCGGTCGTATTATTACGGCTATAAATGTTGCGGCTACTAATCCTATAGGCCTAAATATTGCTGAAAATACCAGCGTCGGTTCTATTGTTACAGATCGTCACTTGTTGCCTGTTAATATTACTGCCGGCAAAAGTTTAACTTTAACCGGTACTGCTGCGTTTGTTCCACGTCATGGTGTTGGTGCTTTTGCTGATACTTATACGGGTTTAGGAAATATAACTTTAGGGGGA | 499 bp; OL687183 |
| **9** | HS63 | AAAATTATTTCAAAAAGCAATACAACAAGGTCTTAAAGCCGCTTTATTCACCACCTCAACCGCAGCGATAATGCTGAGTAGTAGTGGGGTACTCGGTGTTGCTGCAGGTGTTATTGCTACTAATGATGATGCAGCATTTAGTAATGATGCTGAGGCCAATAATTGGGATGAGATAACGGCTGAAGGGGTAGCTAATGGTATTCCTGCTGGCGGTCCTCAAAACAATTGGGCATTTACTTACAGTGCTGATTATACTATCACTGCAGATGTAGTCGATCGTATTATTACGGCTATAAATGTTGCGGGTACTACTCCCGTAGGTCTAAATATTGCTCAAAATACCGTCGTTGGTTCGATTATAACTAGAGGTAACTTGTTGCCTGTTACTATTGCTGGCAAAAGCTTAACTTTAAACGGTACTAATGCTGTTGCTGCAAATCATGGTTTTGATGCTCCGGCCGATAATTATACAGGTTTAGGAAATATAACTTTAGGGGGA | 499 bp; OL687171 |
| **10** | HS69 | AAAATTATTTCAAAAGGCAATTCAAAAAGGTCTTAAAACCGCTTTATTCACCACCTCAACCGCAGCATTAATGCTGAGTAGTAGCGGGGCGTTGGGTGTTGCTGCGGGTGTTATTTCTATTAATAGTGCAGCATTTAGTGATCTTGCTGCTGCCGGTAATTGGAATAAGATAACGGCTGGAGGAGTAGCTAATGGTACTTCTGTTGACGGTCCTCAAGACAATAAGGCATTTACTTACGGTGGTCGTCATATTATCACTGCAGATAAAGTCGGTCGTATTATTACGGCTATAAATGTTGCGGCTACTAATCCTATAGGCCTAAATATTGCTGAAAATACCAGCGTCGGTTCTATTGTTACAGATCGTCACTTGTTGCCTGTTAATATTACTGCCGGCAAAAGTTTAACTTTAACCGGTACTGCTGCGTTTGTTCCACGTCATGGTGTTGGTGCTTTTGCTGATACTTATACGGGTTTAGGAAATATAACTTTAGGGGGA | 499 bp; OL687189 |
| **11** | HS73 | AAAATTATTTCAAAAAGCAATACAACAAGGTCTTAAAGCTGCTTTATTCACCACCTCAACCGCAGCGATAATGCTGAGTAGTAGTGGGGCACTCGGTGTTGCTGCAGGTGTTATTGCTACTAATAATGATGCAGCATTTAGTGATGATGCTGATGTCAATAATTGGGATGAGATAACGGCTATAGGGGTAGCTAATGGTCATCCTGCTGGCGGTCCTCAAAACAATTGGGCATTTACTTACGGTGGTGATTATACTATCACTGCAGATGTAGTCGATTGTATTATTAAGGCTATAAATGTTGCGGGTACTAATCCCGTAGGTCTAAATATTGCTCAAAATACCGTCGTTGGTTCGATTATAACGAGAGGTAACTTGTTGCCTGTTACTATTACTGCCGGCAAAAGCTTAACTTTAAATGGTAATAATGCTGTTGCTGCAAATCATGGTTTTGATGCTCCTGCCGATAATTATACAGGTTTAGGAAATATAACTTTAGGGGGA | 502 bp; OL687166 |
| **12** | HS76 | AAAATTATTTCAAAAAGCAATACAACAAGGTCTTAAAGCCGCTTTATTCACCACCTCAACCGCAGCGATAATGCTGAGTAGTAGTGGGGTACTCGGTGTTGCTGCAGGTGTTATTGCTACTAATGATGATGCAGCATTTAGTAATGATGCTGAGGCCAATAATTGGGATGAGATAACGGCTGAAGGGGTAGCTAATGGTATTCCTGCTGGCGGTCCTCAAAACAATTGGGCATTTACTTACAGTGCTGATTATACTATCACTGCAGATGTAGTCGATCGTATTATTACGGCTATAAATGTTGCGGGTACTACTCCCGTAGGTCTAAATATTGCTCAAAATACCGTCGTTGGTTCGATTATAACTAGAGGTAACTTGTTGCCTGTTACTATTGCTGGCAAAAGCTTAACTTTAAACGGTACTAATGCTGTTGCTGCAAATCATGGTTTTGATGCTCCGGCCGATAATTATACAGGTTTAGGAAATATAACTTTAGGGGGA | 499 bp; OL687172 |
| **13** | HS77 | AAAATTATTTCAAAAGGCAATTCAAAAAGGTCTTAAAACCGCTTTATTCACCACCTCAACCGCAGCATTAATGCTGAGTAGTAGCGGGGCGTTGGGTGTTGCTGCGGGTGTTATTTCTATTAATAGTGCAGCATTTAGTGATCTTGCTGCTGCCGGTAATTGGAATAAGATAACGGCTGGAGGAGTAGCTAATGGTACTTCTGTTGACGGTCCTCAAGACAATAAGGCATTTACTTACGGTGGTCGTCATATTATCACTGCAGATAAAGTCGGTCGTATTATTACGGCTATAAATGTTGCGGCTACTAATCCTATAGGCCTAAATATTGCTGAAAATACCAGCGTCGGTTCTATTGTTACAGATCGTCACTTGTTGCCTGTTAATATTACTGCCGGCAAAAGTTTAACTTTAACCGGTACTGCTGCGTTTGTTCCACGTCATGGTGTTGGTGCTTTTGCTGATACTTATACGGGTTTAGGAAATATAACTTTAGGGGGA | 499 bp; OL687191 |
| **14** | HS81 | AAAATTATTTCAAAAAGCAATACAACAAGGTCTTAAAGCCGCTTTATTCACCACCTCAACCGCAGCGATAATGCTGAGTAGTAGTGGGGTACTCGGTGTTGCTGCAGGTGTTATTGCTACTAATGATGATGCAGCATTTAGTAATGATGCTGAGGCCAATAATTGGGATGAGATAACGGCTGAAGGGGTAGCTAATGGTATTCCTGCTGGCGGTCCTCAAAACAATTGGGCATTTACTTACAGTGCTGATTATACTATCACTGCAGATGTAGTCGATCGTATTATTACGGCTATAAATGTTGCGGGTACTACTCCCGTAGGTCTAAATATTGCTCAAAATACCGTCGTTGGTTCGATTATAACTAGAGGTAACTTGTTGCCTGTTACTATTGCTGGCAAAAGCTTAACTTTAAACGGTACTAATGCTGTTGCTGCAAATCATGGTTTTGATGCTCCGGCCGATAATTATACAGGTTTAGGAAATATAACTTTAGGGGGA | 499 bp; OL687173 |
| **15** | HS122 | AAAATTATTTCAAAAGGCAATTCAAAAAGGTCTTAAAACCGCTTTATTCACCACCTCAACCGCAGCATTAATGCTGAGTAGTAGCGGGGCGTTGGGTGTTGCTGCGGGTGTTATTTCTATTAATAGTGCAGCATTTAGTGATCTTGCTGCTGCCGGTAATTGGAATAAGATAACGGCTGGAGGAGTAGCTAATGGTACTTCTGTTGACGGTCCTCAAGACAATAAGGCATTTACTTACGGTGGTCGTCATATTATCACTGCAGATAAAGTCGGTCGTATTATTACGGCTATAAATGTTGCGGCTACTAATCCTATAGGCCTAAATATTGCTGAAAATACCAGCGTCGGTTCTATTGTTACAGATCGTCACTTGTTGCCTGTTAATATTACTGCCGGCAAAAGTTTAACTTTAACCGGTACTGCTGCGTTTGTTCCACGTCATGGTGTTGGTGCTTTTGCTGATACTTATACGGGTTTAGGAAATATAACTTTAGGGGGA | 499 bp; OL687192 |
| **16** | HS128 | AAAATTATTTCAAAAGGCAATTCAAAAAGGTCTTAAAACCGCTTTATTCACCACCTCAACCGCAGCATTAATGCTGAGTAGTAGCGGGGCGTTGGGTGTTGCTGCGGGTGTTATTTCTATTAATAGTGCAGCATTTAGTGATCTTGCTGCTGCCGGTAATTGGAATAAGATAACGGCTGGAGGAGTAGCTAATGGTACTTCTGTTGACGGTCCTCAAGACAATAAGGCATTTACTTACGGTGGTCGTCATATTATCACTGCAGATAAAGTCGGTCGTATTATTACGGCTATAAATGTTGCGGCTACTAATCCTATAGGCCTAAATATTGCTGAAAATACCAGCGTCGGTTCTATTGTTACAGATCGTCACTTGTTGCCTGTTAATATTACTGCCGGCAAAAGTTTAACTTTAACCGGTACTGCTGCGTTTGTTCCACGTCATGGTGTTGGTGCTTTTGCTGATACTTATACGGGTTTAGGAAATATAACTTTAGGGGGA | 499 bp; OL687184 |
| **17** | HS129 | AAAATTATTTCAAAAAGCAATACAACAAGGTCTTAAAGCCGCTTTATTCACCACCTCAACCGCAGCGATAATGCTGAGTAGTAGTGGGGTACTCGGTGTTGCTGCAGGTGTTATTGCTACTAATGATGATGCAGCATTTAGTAATGATGCTGAGGCCAATAATTGGGATGAGATAACGGCTGAAGGGGTAGCTAATGGTATTCCTGCTGGCGGTCCTCAAAACAATTGGGCATTTACTTACAGTGCTGATTATACTATCACTGCAGATGTAGTCGATCGTATTATTACGGCTATAAATGTTGCGGGTACTACTCCCGTAGGTCTAAATATTGCTCAAAATACCGTCGTTGGTTCGATTATAACTAGAGGTAACTTGTTGCCTGTTACTATTGCTGGCAAAAGCTTAACTTTAAACGGTACTAATGCTGTTGCTGCAAATCATGGTTTTGATGCTCCGGCCGATAATTATACAGGTTTAGGAAATATAACTTTAGGGGGA | 499 bp; OL687174 |
| **18** | H18 | AAAATTATTTCAAAAGGCAATTCAAAAAGGTCTTAAAACCGCTTTATTCACCACCTCAACCGCAGCATTAATGCTGAGTAGTAGCGGGGCGTTGGGTGTTGCTGCGGGTGTTATTTCTATTAATGATGCAGCATTTAGTGATCTTGCTGCTGCCGGTAATTGGAATAAGATAACGGCTGGAGGAGTAGCTAATGGTACTTCTGTTGCCGGTCCTCAAGACAATAAGGCATTTACTTACGGTGGTCGTCATATTATCACTGCAGATAAAGTCGGTCGTATTATTACGGCTATAAATGTTGCGGCTACTAATCCTATAGGCCTAAATATTGCTGAAAATACCAGCGTCGGTTCTATTGTTACAGATCGTAACTTGTTGCCTGTTAATATTAATGCTGGCAAAAGTTTAACTTTAACCGGTACTGCTGCGTTTGTTCCACGTCATGGTGTTGGTGCTTTTGCTGATACTTATACGGGTTTAGGAAATATAACTTTCGGGGGA | 499 bp; OL687176 |
| **19** | H20 | AAAATTATTTCAAAAGGCAATTCAAAAAGGTCTTAAAACTGCTTTATTCACCACCTCAACCGCAGCATTAATGCTGAGTAGTAGCGGGGCGTTGGGTGTTGCTGCGGGTGTTATTTCTACTAATGATGCAGCATTTAGTGATCTTGCTGCTGCCGGTAATTGGAATGAGATAACGGCTGAAGGAGTAGCTAATGGTACTCCTGCTAACGGTCCTCAAGACAATAAGGCATTTACTTACGGTGGTAATCATAATATCACTGCAGATGAAGTCGGTCGTATTATTACGGCTATAAATGTTGCGGCTACTAATCCTGGAGGCCTAAATATTACTGAAGATACCAGCGTCGGTTCTATTGTTACAGGGGGTAACTTGTTGCCTGTTACTATTAATGCCGGCAAAAGTTTAACTTTAACCGGTACTGCTACGGTTGTTGCAAATCATGGTTTTGATGCTGCTGCTGATACTTATACTGGTTTAGGAAATATAACTTTAGGGGGA | 499 bp; OL687194 |
| **20** | H53 | AAAATTATTTCAAAAGGCAATTCAAAAAGGTCTTAAAACCGCTTTATTCACCACCTCAACCGCAGCATTAATGCTGAGTAGTAGCGGGGCGTTGGGTGTTGCTGCGGGTGTTATTTCTATTAATAGTGCAGCATTTAGTGATCTTGCTGCTGCCGGTAATTGGAATAAGATAACGGCTGGAGGAGTAGCTAATGGTACTTCTGTTGACGGTCCTCAAGACAATAAGGCATTTACTTACGGTGGTCGTCATATTATCACTGCAGATAAAGTCGGTCGTATTATTACGGCTATAAATGTTGCGGCTACTAATCCTATAGGCCTAAATATTGCTGAAAATACCAGCGTCGGTTCTATTGTTACAGATCGTCACTTGTTGCCTGTTAATATTACTGCCGGCAAAAGTTTAACTTTAACCGGTACTGCTGCGTTTGTTCCACGTCATGGTGTTGGTGCTTTTGCTGATACTTATACGGGTTTAGGAAATATAACTTTAGGGGGA | 499 bp; OL687193 |
| **21** | H56 | AAAATTATTTCAAAAGGCAATTCAAAAAGGTCTTAAAACCGCTTTATTCACCACCTCAACCGCAGCATTAATGCTGAGTAGTAGCGGGGCGTTGGGTGTTGCTGCGGGTGTTATTTCTATTAATGATGCAGCATTTAGTGATCTTGCTGCTGCCGGTAATTGGAATAAGATAACGGCTGGAGGAGTAGCTAATGGTACTTCTGTTGACGGTCCTCAAGACAATAAGGCATTTACTTACGGTGGTCGTCATATTATCACTGCAGATAAAGTCGGTCGTATTATTACGGCTATAAATGTTGCGGCTACTAATCCTATAGCTATAGGCCTAAATATTGCTGAAAATACCAGCGTCGGTTCTATTGTTACAGATCGTCACTTGTTGCCTGTTAATATTACTGCCGGCAAAAGTTTAACTTTAACCGGTACTGCTGCGTTTGTTCCACGTCATGGTTTTGGTGCTTTTGCTGATACTTATACGGGTTTAGGAAATATAACTTTAGGGGGA | 505 bp; OL687177 |
| **22** | H57 | AAAATTATTTCAAAAGGCAATTCAAAAAGGTCTTAAAACCGCTTTATTCACCACCTCAACCGCAGCATTAATGCTGAGTAGTAGCGGGGCGTTGGGTGTTGCTGCGGGTGTTATTTCTATTAATAGTGCAGCATTTAGTGATCTTGCTGCTGCCGGTAATTGGAATAAGATAACGGCTGGAGGAGTAGCTAATGGTACTTCTGTTGACGGTCCTCAAGACAATAAGGCATTTACTTACGGTGGTCGTCATATTATCACTGCAGATAAAGTCGGTCGTATTATTACGGCTATAAATGTTGCGGCTACTAATCCTATAGGCCTAAATATTGCTGAAAATACCAGCGTCGGTTCT | 352 bp; OL687178 |
| **23** | H75 | AAAATTATTTCAAAAGGCAATTCAAAAAGGTCTTAAAACCGCTTTATTCACCACCTCAACCGCAGCATTAATGCTGAGTAGTAGCGGGGCGTTGGGTGTTGCTGCGGGTGTTATTTCTATTAATAGTGCAGCATTTAGTGATCTTGCTGCTGCCGGTAATTGGAATAAGATAACGGCTGGAGGAGTAGCTAATGGTACTTCTGTTGACGGTCCTCAAGACAATAAGGCATTTACTTACGGTGGTCGTCATATTATCACTGCAGATAAAGTCGGTCGTATTATTACGGCTATAAATGTTGCGGCTACTAATCCTATAGGCCTAAATATTGCTGAAAATACCAGCGTCGGTTCTATTGTTACAGATCGTCACTTGTTGCCTGTTAATATTACTGCCGGCAAAAGTTTAACTTTAACCGGTACTGCTGCGTTTGTTCCACGTCATGGTGTTGGTGCTTTTGCTGATACTTATACGGGTTTAGGAAATATAACTTTAGGGGGA | 499 bp; OL687179 |
| **24** | H78 | AAAATTATTTCAAAAAGCAATACAACAAGGTCTTAAAGCCGCTTTATTCACCACCTCAACCGCAGCGATAATGCTGAGTAGTAGTGGGGTACTCGGTGTTGCTGCAGGTGTTATTGCTACTAATGATGATGCAGCATTTAGTAATGATGCTGAGGCCAATAATTGGGATGAGATAACGGCTGAAGGGGTAGCTAATGGTATTCCTGCTGGCGGTCCTCAAAACAATTGGGCATTTACTTACAGTGCTGATTATACTATCACTGCAGATGTAGTCGATCGTATTATTACGGCTATAAATGTTGCGGGTACTACTCCCGTAGGTCTAAATATTGCTCAAAATACCGTCGTTGGTTCGATTATAACTAGAGGTAACTTGTTGCCTGTTACTATTGCTGGCAAAAGCTTAACTTTAAACGGTACTAATGCTGTTGCTGCAAATCATGGTTTTGATGCTCCGGCCGATAATTATACAGGTTTAGGAAATATAACTTTAGGGGGA | 499 bp; OL687175 |
| **25** | H166 | AAAATTATTTCAAAAGGCAATTCAAAAAGGTCTTAAAACCGCTTTATTCACCACCTCAACCGCAGCATTAATGCTGAGTAGTAGCGGGGCGTTGGGTGTTGCTGCGGGTGTTATTTCTATTAATAGTGCAGCATTTAGTGATCTTGCTGCTGCCGGTAATTGGAATAAGATAACGGCTGGAGGAGTAGCTAATGGTACTTCTGTTGACGGTCCTCAAGACAATAAGGCATTTACTTACGGTGGTCGTCATATTATCACTGCAGATAAAGTCGGTCGTATTATTACGGCTATAAATGTTGCGGCTACTAATCCTATAGGCCTAAATATTGCTGAAAATACCAGCGTCGGTTCTATTGTTACAGATCGTCACTTGTTGCCTGTTAATATTACTGCCGGCAAAAGTTTAACTTTAACCGGTACTGCTGCGTTTGTTCCACGTCATGGTGTTGGTGCTTTTGCTGATACTTATACGGGTTTAGGAAATATAACTTTAGGGGGA | 499 bp; OL687187 |
| **26** | H171 | AAAATTATTTCAAAAGGCAATTCAAAAAGGTCTTAAAACCGCTTTATTCACCACCTCAACCGCAGCATTAATGCTGAGTAGTAGCGGGGCGTTGGGTGTTGCTGCGGGTGTTATTTCTATTAATAGTGCAGCATTTAGTGATCTTGCTGCTGCCGGTAATTGGAATAAGATAACGGCTGGAGGAGTAGCTAATGGTACTTCTGTTGACGGTCCTCAAGACAATAAGGCATTTACTTACGGTGGTCGTCATATTATCACTGCAGATAAAGTCGGTCGTATTATTACGGCTATAAATGTTGCGGCTACTAATCCTATAGGCCTAAATATTGCTGAAAATACCAGCGTCGGTTCTATTGTTACAGATCGTCACTTGTTGCCTGTTAATATTACTGCCGGCAAAAGTTTAACTTTAACCGGTACTGCTGCGTTTGTTCCACGTCATGGTGTTGGTGCTTTTGCTGATACTTATACGGGTTTAGGAAATATAACTTTAGGGGGA | 499 bp; OL687180 |
| **27** | H173 | AAAATTATTTCAAAAGGCAATTCAAAAAGGTCTTAAAACCGCTTTATTCACCACCTCAACCGCAGCATTAATGCTGAGTAGTAGCGGGGCGTTGGGTGTTGCTGCGGGTGTTATTTCTATTAATAGTGCAGCATTTAGTGATCTTGCTGCTGCCGGTAATTGGAATAAGATAACGGCTGGAGGAGTAGCTAATGGTACTTCTGTTGACGGTCCTCAAGACAATAAGGCATTTACTTACGGTGGTCGTCATATTATCACTGCAGATAAAGTCGGTCGTATTATTACGGCTATAAATGTTGCGGCTACTAATCCTATAGGCCTAAATATTGCTGAAAATACCAGCGTCGGTTCTATTGTTACAGATCGTCACTTGTTGCCTGTTAATATTACTGCCGGCAAAAGTTTAACTTTAACCGGTACTGCTGCGTTTGTTCCACGTCATGGTGTTGGTGCTTTTGCTGATACTTATACGGGTTTAGGAAATATAACTTTAGGGGGA | 499 bp; OL687181 |
| **28** | H177 | AAAATTATTTCAAAAGGCAATTCAAAAAGGTCTTAAAACCGCTTTATTCACCACCTCAACCGCAGCATTAATGCTGAGTAGTAGCGGGGCGTTGGGTGTTGCTGCGGGTGTTATTTCTATTAATAGTGCAGCATTTAGTGATCTTGCTGCTGCCGGTAATTGGAATAAGATAACGGCTGGAGGAGTAGCTAATGGTACTTCTGTTGACGGTCCTCAAGACAATAAGGCATTTACTTACGGTGGTCGTCATATTATCACTGCAGATAAAGTCGGTCGTATTATTACGGCTATAAATGTTGCGGCTACTAATCCTATAGGCCTAAATATTGCTGAAAATACCAGCGTCGGTTCTATTGTTACAGATCGTCACTTGTTGCCTGTTAATATTACTGCCGGCAAAAGTTTAACTTTAACCGGTACTGCTGCGTTTGTTCCACGTCATGGTGTTGGTGCTTTTGCTGATACTTATACGGGTTTAGGAAATATAACTTTAGGGGGA | 499 bp; OL687190 |
| **29** | H179 | AAAATTATTTCAAAAAGCAATACAACAAGGTCTTAAAGCCGCTTTATTCACCACCTCAACCGCAGCGATAATGCTGAGTAGTAGTGGGGTACTCGGTGTTGCTGCAGGTGTTATTGCTACTAATGATGATGCAGCATTTAGTAATGATGCTGAGGCCAATAATTGGGATGAGATAACGGCTGAAGGGGTAGCTAATGGTATTCCTGCTGGCGGTCCTCAAAACAATTGGGCATTTACTTACAGTGCTGATTATACTATCACTGCAGATGTAGTCGATCGTATTATTACGGCTATAAATGTTGCGGGTACTACTCCCGTAGGTCTAAATATTGCTCAAAATACCGTCGTTGGTTCGATTATAACTAGAGGTAACTTGTTGCCTGTTACTATTGCTGGCAAAAGCTTAACTTTAAACGGTACTAATGCTGTTGCTGCAAATCATGGTTTTGATGCTCCGGCCGATAATTATACAGGTTTAGGAAATATAACTTTAGGGGGA | 499 bp; OL687168 |
| **30** | H188 | AAAATTATTTCAAAAGGCAATTCAAAAAGGTCTTAAAACCGCTTTATTCACCACCTCAACCGCAGCATTAATGCTGAGTAGTAGCGGGGCGTTGGGTGTTGCTGCGGGTGTTATTTCTATTAATGATGCAGCATTTAGTGATCTTGCTGCTGCCGGTAATTGGAATAAGATAACGGCTGGAGGAGTAGCTAATGGTACTTCTGTTGCCGGTCCTCAAGACAATAAGGCATTTACTTACGGTGGTCGTCATATTATCACTGCAGATAAAGTCGGTCGTATTATTACGGCTATAAATGTTGCGGCTACTAATCCTATAGGCCTAAATATTGCTGAAAATACCAGCGTCGGTTCTATTGTTACAGATCGTAACTTGTTGCCTGTTAATATTAATGCTGGCAAAAGTTTAACTTTAACCGGTACTGCTGCGTTTGTTCCACGTCATGGTGTTGGTGCTTTTGCTGATACTTATACGGGTTTAGGAAATATAACTTTCGGGGGA | 499 bp; OL687182 |

**Supplementary Table S3.** **DNA sequences of *gltA* gene of detected *Rickettsia* spp.**

| **No.** | **Tick pool** | **Sequence (5ꞌ-3ꞌ)** | **Length; NCBI No.** |
| --- | --- | --- | --- |
| **1** | HS28 | AGTGATAAATATGCTTAAAGAAATCGGTAGTTCTGAGTATATTCCTAAATATATAGCTAAAGCTAAGGATAAAAATGATCCATTTAGGTTAATGGGTTTTGGTCATCGTGTATATAAAAACTATGACCCGCGTGCCACAGTACTTAAAGAAACGTGCAAAGAAGTATTAAAGGAACTCGGGCAGCTAGACAACAATCCGCTCTTACAAATAGCAATAGAACTTGAAGCTATCGCTCTTAAAGATGAATATTTTATTGAGAGAAAATTATATCCAAATGTTGATTTTTATTCGGGTATTATCTATAAGGCTATGG | 314 bp; OL687195 |
| **2** | HS29 | AGTGATAAATATGCTTAAAGAAATCGGTAGTTCTGAGTATATTCCTAAATATATAGCTAAAGCTAAGGATAAAAATGATCCATTTAGGTTAATGGGTTTTGGTCATCGTGTATATAAAAACTATGACCCGCGTGCCACAGTACTTAAAGAAACGTGCAAAGAAGTATTAAAGGAACTCGGGCAGCTAGACAACAATCCGCTCTTACAAATAGCAATAGAACTTGAAGCTATCGCTCTTAAAGATGAATATTTTATTGAGAGAAAATTATATCCAAATGTTGATTTTTATTCGGGTATTATCTATAAGGCTATGG | 314 bp; OL687197 |
| **3** | HS38 | AGTGATAAATATGCTTAAAGAAATCGGTAGTTCTGAGAATATCCCTAAATATATAGCTAAAGCTAAGGATAAAAATGATCCGTTTAGGTTAATGGGTTTCGGTCATCGTGTATATAAAAACTATGACCCGCGTGCCGCAGTACTTAAAGAAACGTGCAAAGAAGTATTAAAGGAACTCGAACAGTTAGAAAATAATCCACTTTTACAAATAGCAATAGAACTTGAAGCTATCGCTCTTAAAGATGAATATTTTATTGAGAGAAAATTATATCCAAATGTTGATTTTTATTCAGGTATTATCTATAAAGCTATGG | 314 bp; OL687216 |
| **4** | HS39 | AGTGATAAATATGCTTAAAGAAATCGGTAGTTCTGAGAATATCCCTAAATATATAGCTAAAGCTAAGGATAAAAATGATCCGTTTAGGTTAATGGGTTTCGGTCATCGTGTATATAAAAACTATGACCCGCGTGCCGCAGTACTTAAAGAAACGTGCAAAGAAGTATTAAAGGAACTCGAACAGTTAGAAAATAATCCACTTTTACAAATAGCAATAGAACTTGAAGCTATCGCTCTTAAAGATGAATATTTTATTGAGAGAAAATTATATCCAAATGTTGATTTTTATTCAGGTATTATCTATAAAGCTATGG | 314 bp; OL687217 |
| **5** | HS40 | AGTGATAAATATGCTTAAAGAAATCGGTAGTTCTGAGTATATTCCTCGATATATAGCTAAAGCTAAGGATAAAAATGATCCATTTAGGTTAATGGGTTTTGGTCATCGTGTATATAAAAACTATGACCCGCGTGCCGCAGTACTTAAAGAAACGTGCAAAGAAGTATTAAAGGAACTCGGGCAGCTAGACAACAATCCGCTCTTACAAATAGCAATAGAACTTGAAGCTATCGCTCTTAAAGATGAATATTTTATTGAGAGAAAATTATATCCAAATGTTGATTTTTATTCGGGTATTATCTATAAGGCTATGG | 314 bp; OL687199 |
| **6** | HS44 | AGTGATAAATATGCTTAAAGAAATCGGTAGTTCTGAGAATATCCCTAAATATATAGCTAAAGCTAAGGATAAAAATGATCCGTTTAGGTTAATGGGTTTCGGTCATCGTGTATATAAAAACTATGACCCGCGTGCCGCAGTACTTAAAGAAACGTGCAAAGAAGTATTAAAGGAACTCGAACAGTTAGAAAATAATCCACTTTTACAAATAGCAATAGAACTTGAAGCTATCGCTCTTAAAGATGAATATTTTATTGAGAGAAAATTATATCCAAATGTTGATTTTTATTCAGGTATTATCTATAAAGCTATGG | 314 bp; OL687219 |
| **7** | HS45 | AGTGATAAATATGCTTAAAGAAATCGGTAGTTCTGAGAATATCCCTAAATATATAGCTAAAGCTAAGGATAAAAATGATCCGTTTAGGTTAATGGGTTTCGGTCATCGTGTATATAAAAACTATGACCCGCGTGCCGCAGTACTTAAAGAAACGTGCAAAGAAGTATTAAAGGAACTCGAACAGTTAGAAAATAATCCACTTTTACAAATAGCAATAGAACTTGAAGCTATCGCTCTTAAAGATGAATATTTTATTGAGAGAAAATTATATCCAAATGTTGATTTTTATTCAGGTATTATCTATAAAGCTATGG | 314 bp; OL687220 |
| **8** | HS46 | AGTGATAAATATGCTTAAAGAAATCGGTAGTTCTGAGTATATTCCTCGATATATAGCTAAAGCTAAGGATAAAAATGATCCATTTAGGTTAATGGGTTTTGGTCATCGTGTATATAAAAACTATGACCCGCGTGCCGCAGTACTTAAAGAAACGTGCAAAGAAGTATTAAAGGAACTCGGGCAGCTAGACAACAATCCGCTCTTACAAATAGCAATAGAACTTGAAGCTATCGCTCTTAAAGATGAATATTTTATTGAGAGAAAATTATATCCAAATGTTGATTTTTATTCGGGTATTATCTATAAGGCTATGG | 314 bp; OL687200 |
| **9** | HS59 | AGTGATAAATATGCTTAAAGAAATCGGTAGTTCTGAGAATATCCCTAAATATATAGCTAAAGCTAAGGATAAAAATGATCCGTTTAGGTTAATGGGTTTCGGTCATCGTGTATATAAAAACTATGACCCGCGTGCCGCAGTACTTAAAGAAACGTGCAAAGAAGTATTAAAGGAACTCGAACAGTTAGAAAATAATCCACTTTTACAAATAGCAATAGAACTTGAAGCTATCGCTCTTAAAGATGAATATTTTATTGAGAGAAAATTATATCCAAATGTTGATTTTTATTCAGGTATTATCTATAAAGCTATGG | 314 bp; OL687214 |
| **10** | HS63 | AGTGATAAATATGCTTAAAGAAATCGGTAGTTCTGAGTATATTCCTCGATATATAGCTAAAGCTAAGGATAAAAATGATCCATTTAGGTTAATGGGTTTTGGTCATCGTGTATATAAAAACTATGACCCGCGTGCCGCAGTACTTAAAGAAACGTGCAAAGAAGTATTAAAGGAACTCGGGCAGCTAGACAACAATCCGCTCTTACAAATAGCAATAGAACTTGAAGCTATCGCTCTTAAAGATGAATATTTTATTGAGAGAAAATTATATCCAAATGTTGATTTTTATTCGGGTATTATCTATAAGGCTATGG | 314 bp; OL687201 |
| **11** | HS69 | AGTGATAAATATGCTTAAAGAAATCGGTAGTTCTGAGAATATCCCTAAATATATAGCTAAAGCTAAGGATAAAAATGATCCGTTTAGGTTAATGGGTTTCGGTCATCGTGTATATAAAAACTATGACCCGCGTGCCGCAGTACTTAAAGAAACGTGCAAAGAAGTATTAAAGGAACTCGAACAGTTAGAAAATAATCCACTTTTACAAATAGCAATAGAACTTGAAGCTATCGCTCTTAAAGATGAATATTTTATTGAGAGAAAATTATATCCAAATGTTGATTTTTATTCAGGTATTATCTATAAAGCTATGG | 314 bp; OL687221 |
| **12** | HS73 | AGTGATAAATATGCTTAAAGAAATCGGTAGTTCTGAGTATATTCCTAAATATATAGCTAAAGCTAAGGATAAAAATGATCCATTTAGGTTAATGGGTTTTGGTCATCGTGTATATAAAAACTATGACCCGCGTGCCACAGTACTTAAAGAAACGTGCAAAGAAGTATTAAAGGAACTCGGGCAGCTAGACAACAATCCGCTCTTACAAATAGCAATAGAACTTGAAGCTATCGCTCTTAAAGATGAATATTTTATTGAGAGAAAATTATATCCAAATGTTGATTTTTATTCGGGTATTATCTATAAGGCTATGG | 314 bp; OL687196 |
| **13** | HS76 | AGTGATAAATATGCTTAAAGAAATCGGTAGTTCTGAGTATATTCCTCGATATATAGCTAAAGCTAAGGATAAAAATGATCCATTTAGGTTAATGGGTTTTGGTCATCGTGTATATAAAAACTATGACCCGCGTGCCGCAGTACTTAAAGAAACGTGCAAAGAAGTATTAAAGGAACTCGGGCAGCTAGACAACAATCCGCTCTTACAAATAGCAATAGAACTTGAAGCTATCGCTCTTAAAGATGAATATTTTATTGAGAGAAAATTATATCCAAATGTTGATTTTTATTCGGGTATTATCTATAAGGCTATGG | 314 bp; OL687202 |
| **14** | HS77 | AGTGATAAATATGCTTAAAGAAATCGGTAGTTCTGAGAATATCCCTAAATATATAGCTAAAGCTAAGGATAAAAATGATCCGTTTAGGTTAATGGGTTTCGGTCATCGTGTATATAAAAACTATGACCCGCGTGCCGCAGTACTTAAAGAAACGTGCAAAGAAGTATTAAAGGAACTCGAACAGTTAGAAAATAATCCACTTTTACAAATAGCAATAGAACTTGAAGCTATCGCTCTTAAAGATGAATATTTTATTGAGAGAAAATTATATCCAAATGTTGATTTTTATTCAGGTATTATCTATAAAGCTATGG | 314 bp; OL687223 |
| **15** | HS81 | AGTGATAAATATGCTTAAAGAAATCGGTAGTTCTGAGTATATTCCTCGATATATAGCTAAAGCTAAGGATAAAAATGATCCATTTAGGTTAATGGGTTTTGGTCATCGTGTATATAAAAACTATGACCCGCGTGCCGCAGTACTTAAAGAAACGTGCAAAGAAGTATTAAAGGAACTCGGGCAGCTAGACAACAATCCGCTCTTACAAATAGCAATAGAACTTGAAGCTATCGCTCTTAAAGATGAATATTTTATTGAGAGAAAATTATATCCAAATGTTGATTTTTATTCGGGTATTATCTATAAGGCTATGG | 314 bp; OL687203 |
| **16** | HS83 | AGTGATAAATATGCTTAAAGAAATCGGTAGTTCTGAGTATATTCCTAAATATATAGCTAAAGCTAAGGATAAAAATGATCCATTTAGGTTAATGGGTTTTGGTCATCGTGTATATAAAAACTATGACCCGCGTGCCGCAGTACTGAAAGAAACGTGCAAAGAAGTATTAAAGGAACTCGGGCAGCTAGACAACAATCCGCTCTTACAAATAGCAATAGAACTTGAAGCTATCGCTCTTAAAGATGAATATTTTATTGAGAGAAAATTATATCCAAATGTTGATTTTTATTCGGGTATTATCTATAACGCTATGG | 314 bp; OL687226 |
| **17** | HS122 | AGTGATAAATATGCTTAAAGAAATCGGTAGTTCTGAGAATATCCCTAAATATATAGCTAAAGCTAAGGATAAAAATGATCCGTTTAGGTTAATGGGTTTCGGTCATCGTGTATATAAAAACTATGACCCGCGTGCCGCAGTACTTAAAGAAACGTGCAAAGAAGTATTAAAGGAACTCGAACAGTTAGAAAATAATCCACTTTTACAAATAGCAATAGAACTTGAAGCTATCGCTCTTAAAGATGAATATTTTATTGAGAGAAAATTATATCCAAATGTTGATTTTTATTCAGGTATTATCTATAAAGCTATGG | 314 bp; OL687224 |
| **18** | HS128 | AGTGATAAATATGCTTAAAGAAATCGGTAGTTCTGAGAATATCCCTAAATATATAGCTAAAGCTAAGGATAAAAATGATCCGTTTAGGTTAATGGGTTTCGGTCATCGTGTATATAAAAACTATGACCCGCGTGCCGCAGTACTTAAAGAAACGTGCAAAGAAGTATTAAAGGAACTCGAACAGTTAGAAAATAATCCACTTTTACAAATAGCAATAGAACTTGAAGCTATCGCTCTTAAAGATGAATATTTTATTGAGAGAAAATTATATCCAAATGTTGATTTTTATTCAGGTATTATCTATAAAGCTATGG | 314 bp; OL687215 |
| **19** | HS129 | AGTGATAAATATGCTTAAAGAAATCGGTAGTTCTGAGTATATTCCTCGATATATAGCTAAAGCTAAGGATAAAAATGATCCATTTAGGTTAATGGGTTTTGGTCATCGTGTATATAAAAACTATGACCCGCGTGCCGCAGTACTTAAAGAAACGTGCAAAGAAGTATTAAAGGAACTCGGGCAGCTAGACAACAATCCGCTCTTACAAATAGCAATAGAACTTGAAGCTATCGCTCTTAAAGATGAATATTTTATTGAGAGAAAATTATATCCAAATGTTGATTTTTATTCGGGTATTATCTATAAGGCTATGG | 314 bp; OL687204 |
| **20** | H18 | AGTGATAAATATGCTTAAAGAAATCGGTAGTTCTGAGAATATCCCTAAATATATAGCTAAAGCTAAGGATAAAAATGATCCGTTTAGGTTAATGGGTTTCGGTCATCGTGTATATAAAAACTATGACCCGCGTGCCGCAGTACTTAAAGAAACGTGCAAAGAAGTATTAAAGGAACTCGAACAGTTAGAAAATAATCCACTTTTACAAATAGCAATAGAACTTGAAGCTATCGCTCTTAAAGATGAATATTTTATTGAGAGAAAATTATATCCAAATGTTGATTTTTATTCAGGTATTATCTATAAAGCTATGG | 314 bp; OL687206 |
| **21** | H20 | AGTGATAAATATGCTTAAAGAAATCGGTAGTTCTGAGAATATCCCTAAATATATAGCTAAAGCTAAGGATAAAAATGATCCGTTTAGGTTAATGGGTTTCGGTCATCGTGTATATAAAAACTATGACCCGCGTGCCGCAGTACTTAAAGAAACGTGCAAAGAAGTGTTAAAGGAACTCGGACAGTTAGAAAATAATCCACTTTTACAAATAGCAATAGAACTTGAAGCTATCGCTCTTAAAGATGAATATTTTATTGAGAGAAAATTATATCCAAATGTTGATTTTTATTCAGGTATTATCTATAAAGCTATGG | 314 bp; OL687228 |
| **22** | H53 | AGTGATAAATATGCTTAAAGAAATCGGTAGTTCTGAGAATATCCCTAAATATATAGCTAAAGCTAAGGATAAAAATGATCCGTTTAGGTTAATGGGTTTCGGTCATCGTGTATATAAAAACTATGACCCGCGTGCCGCAGTACTTAAAGAAACGTGCAAAGAAGTATTAAAGGAACTCGAACAGTTAGAAAATAATCCACTTTTACAAATAGCAATAGAACTTGAAGCTATCGCTCTTAAAGATGAATATTTTATTGAGAGAAAATTATATCCAAATGTTGATTTTTATTCAGGTATTATCTATAAAGCTATGG | 314 bp; OL687225 |
| **23** | H56 | AGTGATAAATATGCTTAAAGAAATCGGTAGTTCTGAGAATATCCCTAAATATATAGCTAAAGCTAAGGATAAAAATGATCCGTTTAGGTTAATGGGTTTCGGTCATCGTGTATATAAAAACTATGACCCGCGTGCCGCAGTACTTAAAGAAACGTGCAAAGAAGTATTAAAGGAACTCGAACAGTTCGAAAATAATCCACTTTTACAAATAGCAATAGAACTTGAAGCTATCGCTCTTAAAGATGAATATTTTATTGAGAGAAAATTATATCCAAATGTTGATTTTTATTCAGGTATTATCTATAAAGCTATGG | 314 bp; OL687207 |
| **24** | H57 | AGTGATAAATATGCTTAAAGAAATCGGTAGTTCTGAGAATATCCCTAAATATATAGCTAAAGCTAAGGATAAAAATGATCCGTTTAGGTTAATGGGTTTCGGTCATCGTGTATATAAAAACTATGACCCGCGTGCCGCAGTACTTAAAGAAACGTGCAAAGAAGTATTAAAGGAACTCGAACAGTTAGAAAATAATCCACTTTTACAAATAGCAATAGAACTTGAAGCTATCGCTCTTAAAGATGAATATTTTATTGAGAGAAAATTATATCCAAATGTTGATTTTTATTCAGGTATTATCTATAAAGCTATGG | 314 bp; OL687208 |
| **25** | H75 | AGTGATAAATATGCTTAAAGAAATCGGTAGTTCTGAGAATATCCCTAAATATATAGCTAAAGCTAAGGATAAAAATGATCCGTTTAGGTTAATGGGTTTCGGTCATCGTGTATATAAAAACTATGACCCGCGTGCCGCAGTACTTAAAGAAACGTGCAAAGAAGTATTAAAGGAACTCGAACAGTTAGAAAATAATCCACTTTTACAAATAGCAATAGAACTTGAAGCTATCGCTCTTAAAGATGAATATTTTATTGAGAGAAAATTATATCCAAATGTTGATTTTTATTCAGGTATTATCTATAAAGCTATGG | 314 bp; OL687209 |
| **26** | H78 | AGTGATAAATATGCTTAAAGAAATCGGTAGTTCTGAGTATATTCCTCGATATATAGCTAAAGCTAAGGATAAAAATGATCCATTTAGGTTAATGGGTTTTGGTCATCGTGTATATAAAAACTATGACCCGCGTGCCGCAGTACTTAAAGAAACGTGCAAAGAAGTATTAAAGGAACTCGGGCAGCTAGACAACAATCCGCTCTTACAAATAGCAATAGAACTTGAAGCTATCGCTCTTAAAGATGAATATTTTATTGAGAGAAAATTATATCCAAATGTTGATTTTTATTCGGGTATTATCTATAAGGCTATGG | 314 bp; OL687205 |
| **27** | H166 | AGTGATAAATATGCTTAAAGAAATCGGTAGTTCTGAGAATATCCCTAAATATATAGCTAAAGCTAAGGATAAAAATGATCCGTTTAGGTTAATGGGTTTCGGTCATCGTGTATATAAAAACTATGACCCGCGTGCCGCAGTACTTAAAGAAACGTGCAAAGAAGTATTAAAGGAACTCGAACAGTTAGAAAATAATCCACTTTTACAAATAGCAATAGAACTTGAAGCTATCGCTCTTAAAGATGAATATTTTATTGAGAGAAAATTATATCCAAATGTTGATTTTTATTCAGGTATTATCTATAAAGCTATGG | 314 bp; OL687218 |
| **28** | H167 | AGTGATAAATATGCTTAAAGAAATCGGTAGTTCTGAGAATATCCCTAAATATATAGCTAAAGCTAAGGATAAAAATGATCCGTTTAGGTTAATGGGTTTCGGTCATCGTGTATATAAAAACTATGACCCGCGTGCCGCAGTACTTAAAGAAACGTGCAAAGAAGTATTAAAGGAACTCGAACAGTTAGAAAATAATCCACTTTTACAAATAGCAATAGAACTTGAAGCTATCGCTCTTAAAGATGAATATTTTATTGAGAGAAAATTATATCCAAATGTTGATTTTTATTCAGGTATTATCTATAAAGCTATGG | 314 bp; OL687210 |
| **29** | H171 | AGTGATAAATATGCTTAAAGAAATCGGTAGTTCTGAGAATATCCCTAAATATATAGCTAAAGCTAAGGATAAAAATGATCCGTTTAGGTTAATGGGTTTCGGTCATCGTGTATATAAAAACTATGACCCGCGTGCCGCAGTACTTAAAGAAACGTGCAAAGAAGTATTAAAGGAACTCGAACAGTTAGAAAATAATCCACTTTTACAAATAGCAATAGAACTTGAAGCTATCGCTCTTAAAGATGAATATTTTATTGAGAGAAAATTATATCCAAATGTTGATTTTTATTCAGGTATTATCTATAAAGCTATGG | 314 bp; OL687211 |
| **30** | H173 | AGTGATAAATATGCTTAAAGAAATCGGTAGTTCTGAGAATATCCCTAAATATATAGCTAAAGCTAAGGATAAAAATGATCCGTTTAGGTTAATGGGTTTCGGTCATCGTGTATATAAAAACTATGACCCGCGTGCCGCAGTACTTAAAGAAACGTGCAAAGAAGTATTAAAGGAACTCGAACAGTTAGAAAATAATCCACTTTTACAAATAGCAATAGAACTTGAAGCTATCGCTCTTAAAGATGAATATTTTATTGAGAGAAAATTATATCCAAATGTTGATTTTTATTCAGGTATTATCTATAAAGCTATGG | 314 bp; OL687212 |
| **31** | H177 | AGTGATAAATATGCTTAAAGAAATCGGTAGTTCTGAGAATATCCCTAAATATATAGCTAAAGCTAAGGATAAAAATGATCCGTTTAGGTTAATGGGTTTCGGTCATCGTGTATATAAAAACTATGACCCGCGTGCCGCAGTACTTAAAGAAACGTGCAAAGAAGTATTAAAGGAACTCGAACAGTTAGAAAATAATCCACTTTTACAAATAGCAATAGAACTTGAAGCTATCGCTCTTAAAGATGAATATTTTATTGAGAGAAAATTATATCCAAATGTTGATTTTTATTCAGGTATTATCTATAAAGCTATGG | 314 bp; OL687222 |
| **32** | H179 | AGTGATAAATATGCTTAAAGAAATCGGTAGTTCTGAGTATATTCCTCGATATATAGCTAAAGCTAAGGATAAAAATGATCCATTTAGGTTAATGGGTTTTGGTCATCGTGTATATAAAAACTATGACCCGCGTGCCGCAGTACTTAAAGAAACGTGCAAAGAAGTATTAAAGGAACTCGGGCAGCTAGACAACAATCCGCTCTTACAAATAGCAATAGAACTTGAAGCTATCGCTCTTAAAGATGAATATTTTATTGAGAGAAAATTATATCCAAATGTTGATTTTTATTCGGGTATTATCTATAAGGCTATGG | 314 bp; OL687198 |
| **33** | H188 | AGTGATAAATATGCTTAAAGAAATCGGTAGTTCTGAGAATATCCCTAAATATATAGCTAAAGCTAAGGATAAAAATGATCCGTTTAGGTTAATGGGTTTCGGTCATCGTGTATATAAAAACTATGACCCGCGTGCCGCAGTACTTAAAGAAACGTGCAAAGAAGTATTAAAGGAACTCGAACAGTTAGAAAATAATCCACTTTTACAAATAGCAATAGAACTTGAAGCTATCGCTCTTAAAGATGAATATTTTATTGAGAGAAAATTATATCCAAATGTTGATTTTTATTCAGGTATTATCTATAAAGCTATGG | 314 bp; OL687213 |
| **34** | H194 | AGTGATAAATATGCTTAAAGAAATCGGTAGTTCTGAGTATATTCCTAAATATATAGCTAAAGCTAAGGATAAAAATGATCCATTTAGGTTAATGGGTTTTGGTCATCGTGTATATAAAAAACTATGACCCGCGTGCCGCAGTACTGAAAGAAACGTGCAAAGAAGTATTAAAGGAACTCGGGCAGCTAGACAACAATCCGCTCTTACAAATAGCAATAGAACTTGAAGCTATCGCTCTTAAAGATGAATATTTTATTGAGAGAAAATTATATCCAAATGTTGATTTTTATTCGGGTATTATCTATAACGCTATGG | 314 bp; OL687227 |


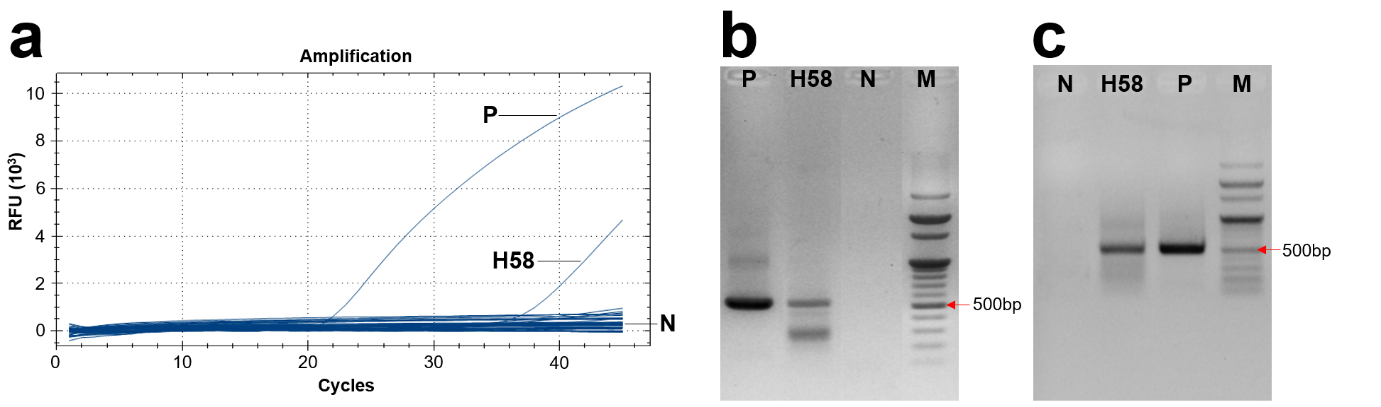


**Supplementary Figure S1. Detection of *Toxoplasma gondii* from bird ticks.** (**A**) *T. gondii* detected from tick sample H58 using real-time PCR. (**B**) The H58 sample was also used for conventional PCR and (**C**) in nested PCR. Positive results were confirmed with expected band of 529 bp and 504 bp long, respectively. “P” and “N” indicate positive control using *T. gondii* DNA and negative control (N) without DNA template, respectively. “M” is 100bp DNA marker.


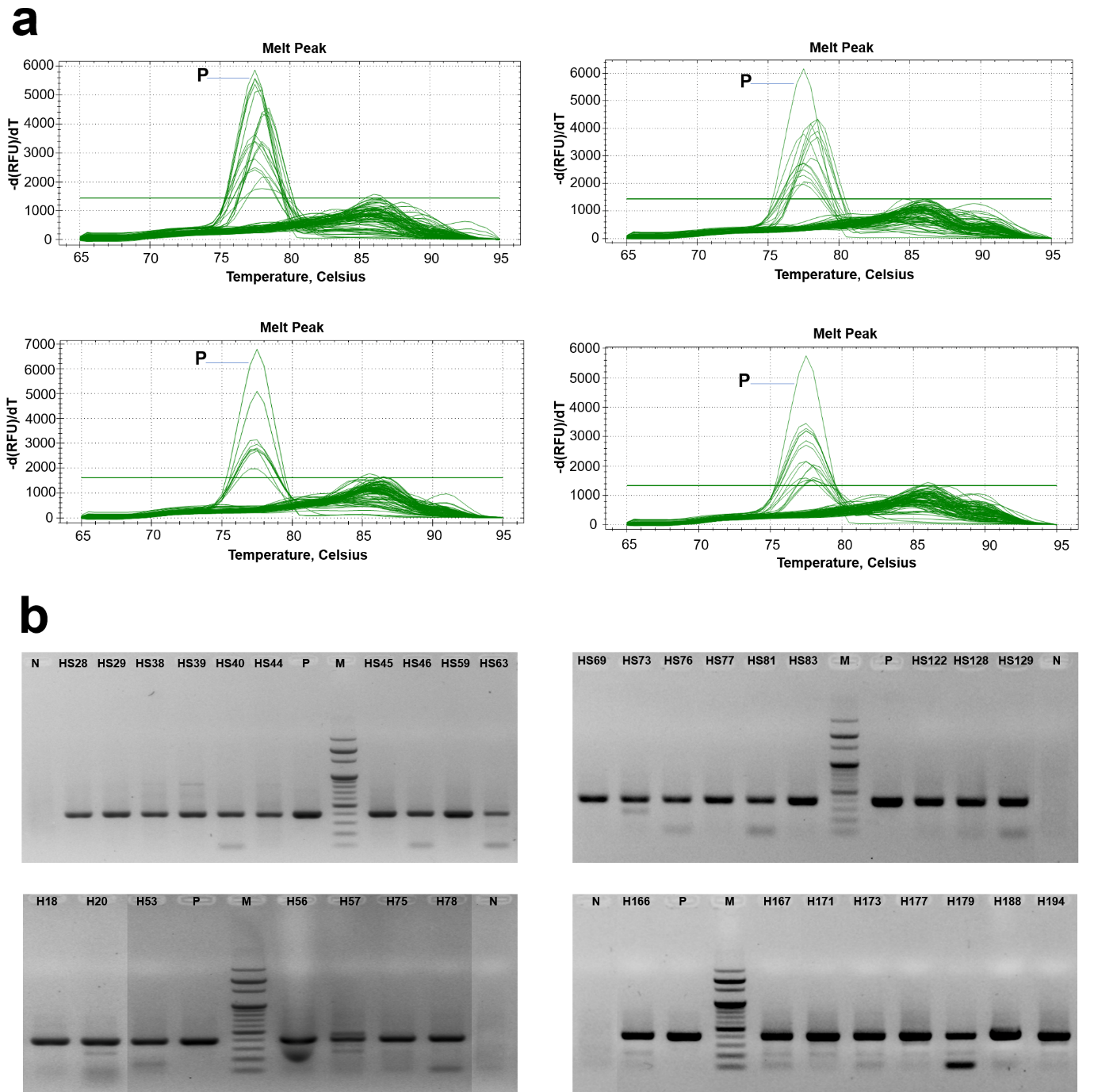


**Supplementary Figure S2. Detection of *Rickettsia* spp. from ticks collected from migratory birds.** Detection of *Rickettsia* spp. was conducted by comparing melting curve peak with the positive control (P) using recombinant *R. japonica* DNA*.* A total of 34 pools of ticks were positive using real-time PCR and were confirmed using gel electrophoresis with expected band of 388 bp long. The negative control “N” without DNA template, and “M” 100 bp DNA marker.
